# Understanding memristive switching via in situ characterization and device modeling

**DOI:** 10.1038/s41467-019-11411-6

**Published:** 2019-08-01

**Authors:** Wen Sun, Bin Gao, Miaofang Chi, Qiangfei Xia, J. Joshua Yang, He Qian, Huaqiang Wu

**Affiliations:** 1 Institute of Microelectronics, Tsinghua University, 100084 Beijing, China; 2 Center for Nanophase Materials Sciences, Oak Ridge National Laboratory, One Bethel Valley Road, Building 4515, Oak Ridge, TN 37831-6064 USA; 3 Department of Electrical and Computer Engineering, University of Massachusetts, Amherst, MA 01003-9292 USA

**Keywords:** Electronic devices, Characterization and analytical techniques

## Abstract

Owing to their attractive application potentials in both non-volatile memory and unconventional computing, memristive devices have drawn substantial research attention in the last decade. However, major roadblocks still remain in device performance, especially concerning relatively large parameter variability and limited cycling endurance. The response of the active region in the device within and between switching cycles plays the dominating role, yet the microscopic details remain elusive. This Review summarizes recent progress in scientific understanding of the physical origins of the non-idealities and propose a synergistic approach based on in situ characterization and device modeling to investigate switching mechanism. At last, the Review offers an outlook for commercialization viability of memristive technology.

## Abstract (web summary)

Memristor as the fourth basic element of electric circuits has drawn substantial attention for developing future computing technologies. Sun et al. report the progress and the challenges facing researchers on understanding memristive switching, and advocate continuous studies using a synergistic approach.

## Introduction

Memristive devices have been studied intensively since the link between memristor theory and physical resistive switching devices was established in 2008^1^, which was initially driven by the need for high-performance non-volatile memory and has more recently been fueled by energy-efficient unconventional computing^2^. Postulated as the fourth fundamental passive circuit element in addition to resistor, capacitor, and inductor, the memristor can store information in a form of resistance, which can be modulated by the history of its external stimuli^3^. The typical structure of memristors is a two-terminal three-layered stack, consisting of a switching layer sandwiched between two metallic electrodes. The switching layer ranges from semiconducting to insulating inorganic or organic materials. Through materials engineering, memristive devices can be tailored to provide non-volatile or volatile memory. Non-volatile memristor maintains its resistance state after the removal of the applied switching voltage or current. The stable resistance state is used to represent stored information, making memristive devices suitable for data storage applications. Integrated memristive crossbar arrays are considered promising candidates for future application in mainstream non-volatile memory. This is because memristive devices can store information at the sub-2-nm scale^4^ and possess many other desired properties, including high speed^5^, low energy consumption, three-dimensional integration capability, and compatibility with complementary metal oxide semiconductor (CMOS) technologies^6^. Furthermore, in-memory analog computing is being developed to process information where it is stored^7^. Such in-memory computing is expected to offer an efficient and reconfigurable solution to process analog information in artificial intelligence (AI) applications^8^. On the other hand, the programmed resistance state of a volatile memristor gradually relaxes toward a thermodynamically stable state upon the removal of the programming signal, offering desirable dynamics for emulating biological synapses and neurons^9^. Memristors at the individual device level have shown short- and long-term plasticity similar to that of biological synapses^9,10^. Neural networks with memristors at the array level have been used to demonstrate brain-inspired functions^11^.

**Fig. 1:**
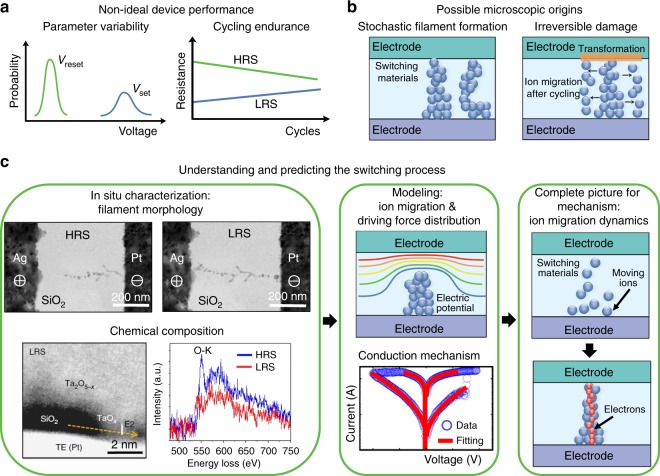
Synergistic approaches for mechanistic research of memristive devices for improving the device performance. **a** Parameter variability of the set and reset voltages (left), and typical endurance failure behaviors in memristors (right). HRS represents the high resistance state, and LRS represents the low resistance state. **b** Possible microscopic origins responsible for the device behaviors: conduction filaments in different forms contribute to the parameter variability (left of panel **a**); after cycling operation, the formation of an interfacial layer with high series resistance (orange disc), or the expansion of migration area for mobile ions (migrated blue balls marked by black arrows), leading to the cycling endurance failure (right of panel **a**). **c** The in situ characterizations and device modeling can complement and complete each other and together provide a holistic picture of the resistive switching, as shown in the middle where a device model is schematically presented with switching *I*–*V* loops of simulated and experimental data. Reproduced from ref. ^12^, Macmillan Publishers Ltd (**c**), Ag/SiO_2_/Pt device; ref. ^22^, Macmillan Publishers Ltd (**c**), Ta_2_O_5-x_/SiO_2_/Pt device

Consequently, memristive devices have attracted significant attention in the past decade as a key enabler of new computing paradigms to overcome the limitations of the conventional von Neumann computing architecture. However, although significant research efforts have been directed toward memristive devices, a large-scale commercialization of these devices has not yet been achieved. In addition to challenges at the circuit, algorithm and architecture levels, issues at the device level are likely still the primary reason. Two of the major remaining challenges at the device level are relatively large parameter variability and poor cycling endurance (Fig. 1a). To improve the cycling endurance and reduce the parameter variability to a level sufficient for large-scale commercialization, it is necessary to acquire an in-depth understanding of ion migration and its coupling with electron transport—the dominating dynamics in memristive mechanism—during the switching process. To reveal the dynamic process of switching, in situ characterization techniques are necessary; then, device modeling is needed to thoroughly explain the phenomena observed in situ. As such, in situ characterization combined with device modeling is the most efficient approach to achieve a complete and in-depth understanding of the switching mechanism. In this Review, we summarize state-of-the-art understanding of memristive switching mechanism and discuss future research directions. We primarily focus on in situ characterization techniques and device modeling methods, aiming to monitor and analyze the switching behavior of memristors at the single-atom level.

## Memristive switching mechanism

Based on the active switching region and geometry, memristors can be classified as filament type, interface type, and bulk type. Among them, the filament type memristors have been attracted more research attentions due to their overall superior performance as well as greater challenges in mechanism studies. Thus, the Review will discuss more on filament type memristors than other types. On the other hand, according to the type of mobile species and migration behavior, memristors can be classified as cation devices, anion devices, and dual ionic devices (Box 1). To understand the underlying memristive switching mechanisms, the current research has been focused on the following five aspects: chemical composition of materials in the active region; driving forces for ion migration; filament morphology; electron conduction mechanisms; and switching dynamics (Table 1). It is notable that a complete mechanistic understanding of resistive switching still remains largely ambiguous at this stage due to limited assessable experimental data, especially those concerning switching dynamics, such as the evolution of filament morphology and transport mechanism during repetitive cycling.

**Table 1:** The research status on switching mechanism of memristive devices

Properties	Common items for all types	Cation devices	Anion devices	Dual ionic devices
Chemical composition	Ion migration or phase change	Active metals, such as Ag, Cu *Less active metals, such as Ti, Ta*	Oxygen ions or vacancies *Other anions, such as nitrogen vacancies*	*Both cation and anion*
Driving force	Electric field *Thermal effects (thermophoresis)*^20^ *Chemical potential gradient*^92^	*Nanobattery effect*^93^ *Interfacial energy minimization*^9^	*Relative role of field and temperature* *Thermodynamics*^2^	*Relative role of field and temperature* *Thermodynamics*
Filament morphology	Filamentary *Single and multiple filaments* *Dendrite-like filament*^45^ *Inverted or forward cone shape*^14^	*Chain of nanoparticles*^12^ *Inverted triangle crystalline*^13^ *Non-filamentary*^81^	Non-filamentary^94^ *Percolation path* *Filament rupture region*	*Percolation path* *Filament rupture region*
Electron conduction mechanism	Ohmic conduction Schottky emission Tunneling (direct or FN)	*SCLC model*^95^ *Quantum conductance*^96^	*P-F model, SCLC model*^97^ *Hopping (fixed-ranged, variable-ranged)*^98^ *TAT model*	*Trap Assisted Tunneling (TAT) model*
Switching dynamics	*Redox reaction* *Nucleation*^99^ *Microscopic picture of switching*	*Filament growth direction* *Growth dynamics*^14^ *Filament dissolution*	*Oxygen vacancy generation in the bulk* *Interstitial oxygen ion migration* *Dynamic motion of oxygen*	*Migration dynamics of cations and anions* *Reaction of cations and anions*

Non-italicized indicates the conclusive findings, and italicized represents arguments not fully conclusive yet

Box 1 Classification of memristive switching mechanisms During memristive switching, the atomic motion induces microscopic changes in memristors following three types of reconfiguration. The first type is filamentary switching, the mechanism of which is correlated with the growth of single or multiple localized conduction filaments through the switching layer^80^. This type is very common, and the active switching region is concentrated at the tip of a filament typically close to the interface between the switching layer and an electrode. The second type is non-filamentary interfacial switching, in which resistive switching is resulted from the modulation of the carrier transport barrier at the electrode/switching layer interface induced by ion migration^81,82^. This type of switching is typically slow, likely because of the lack of effective Joule heating associated with a localized conduction filament to induce a local temperature increase and to enable a fast ion motion at elevated temperature during switching. The third type of resistive switching occurs inside the bulk of the entire or a part of the switching layer, which is typically triggered by a phase change, and it involves short-term atomic rearrangement. The phase change devices include an amorphous-crystalline transition as in the Ge_x_Sb_y_Te_z_ memristors (also called phase change memory, PCM)^83^, and topological transition or lattice structure transition as in VO_2_ and NbO_2_ memristors (also called Mott memristors)^27^.

According to the types of mobile species responsible for the resistive switching, memristors can be classified into three categories, namely cation devices (also called electrochemical metallization memory, ECM), anion devices (also called valence charge memory, VCM), and dual ionic devices. A typical cation device consists of an electrochemically active metal/dielectric/inert metal structure. When the scale is reduced to nano-size, the dielectric layer can be converted into solid electrolytes^84^. The mobile ions are usually the cations of the active metal, such as Ag, Cu, Ni, or an alloy of these metals^14,15^. In anion devices, the switching layers are made of compound or organic materials, including binary metal oxides, complex oxides, carbon-based oxides, nitrides, chalcogenides, etc^2^. Among them, metal oxides are the most common material system, in which the mobile ions are normally oxygen ions, or equivalently, oxygen vacancies^6^. Recently, a multi-switching mechanism involving the motion of both cations and anions has been established by a number of reports^30,33^, and thus, these memristors can be defined as dual ionic devices.

Schematics of switching mechanisms using different classification criteria: **a** Filamentary switching. **b** Interfacial switching. **c** Bulk switching caused by phase change. **d** Set operation of Cation device: Ag/SiO_2_/Pt. **e** Set operation of Anion device: TiN/HfO_2_/Pt. **f** Set operation of Dual ionic devices: Ag/Ta_2_O_5_/Pt. During set operation, positive voltage is applied on the top electrodes, and the conduction filament is marked by a black dashed line in **d**–**f**.

## Cation memristors

Among the active electrode metals in cation devices, Ag has been studied intensively by in situ transmission electron microscopy (TEM) for understanding the switching mechanism of memristive devices. During electroforming, the growth of conduction filaments has been observed in the form of a chain of Ag nanoparticles^12^ or an Ag crystalline phase^13^. The growth direction and switching dynamics of conduction filaments are governed by kinetic factors, such as the ion mobility and the redox rate^14,15^. Under the driving force of electric field, the migration of Ag nanoparticles follows a step-by-step mass transport process to form a complete conduction filament^16^.Under the reverse voltage, the conduction filaments are ruptured and a gap region is formed. In volatile cation memristor, which is also called diffusion memristor, the Ag filament relaxed into a round shape to minimize the interfacial energy after the removal of the bias^9^. Ag or Cu can function as the active electrode for both non-volatile and volatile memristors. Whether or not they maintain the conduction filaments after set operations without external bias depends on the parameters defining the system, such as the diameter of the conduction channel, the interfacial energy between the channel and dielectric materials, the heat dissipation, and local temperature, and others^17^.

## Anion memristors

The oxygen migration in anion devices is important because it is involved in the switching mechanism of various materials systems. In fact, the conduction path in anion device is a chemically reduced metal with a lower valence state, which can be regarded as being composed of oxygen vacancies as n-type dopants. In memristors made up of complex oxides with a single-crystalline structure, such as Pr_0.7_Ca_0.3_MnO_3_^18^, BiFeO_3_^19^, and La_2/3_Sr_1/3_MnO_3_^20^, the dynamic behavior of oxygen ions/vacancies has been clarified with conclusive experimental evidence because oxygen vacancies can be observed directly. In these single-crystalline materials, the oxygen vacancies originate from the crystalline switching layer^19^. On the other hand, the memristors with amorphous oxide structures, such as CuO^21^, TaO_x_^22^, Al_2_O_3_^23^, HfO_2_^24^, NiO^25^, TiO_2_^26^, NbO_2_^27^, MoO_x_^28^, and ZnO^29^, have great advantages in terms of their fabrication cost, fab compatibility and device performance. The conclusions regarding the origination and dynamic activities of oxygen vacancies in these amorphous oxides remain controversial, primarily owing to the challenge of direct observation of oxygen vacancies. Various driving forces, conductance mechanisms and switching dynamics were proposed, but only a few of them are conclusive.

## Dual ionic memristors

In 2015, Wedig et al. first speculated that cation migration of Ta, Hf, and Ti ions could dominate the resistive switching in metal oxide memristors, competing with the migration of oxygen vacancies^30^. Since then, a multi-switching mechanism involving the motion of both cations and anions has been experimentally observed. In these dual ionic devices, the motion of cations or/and anions can play the dominating role in the switching process, depending on the migration energy barrier of mobile species in the switching layer^31^, such as oxygen vacancies in Ni/HfO_2_/SiO_2_/Si^32^, Ag ion in Ag/Ta_2_O_5_/Pt^33^, and simultaneously migration of Ta ions and oxygen vacancies in Ta/HfO_2_/Pt^31^. In addition, noble metal ions can also act as migrating cations, such as the Pd ions in a SiO_2_ switching layer^34^. As a result of the cation movement towards the cathode and the anion movement towards the anode, the conduction filaments can be identified as metal atoms^31,35^ or a combination of metal atoms and oxygen vacancies^33^. However, the dynamic evolution of cations and anions needs further explorations to reveal the details.

## The importance of understanding switching mechanism

The fundamental reasons for the non-ideal performance of memristive devices, including the large parameter variability, cycling endurance degradation, and so on, are attributed to uncontrolled ion migration. For example, in the filament type memristor, the ion migration dominates the evolution of conduction filaments. The initial formation of conduction filaments decides the device-to-device variability, while the repeated rupture and re-formation of the filaments affect the cycle-to-cycle variability and cycling endurance (Fig. 1a, b). Parameter variability and cycling endurance issues are not concerns for metal oxide semiconductor (MOS) transistors because they rely on the transport and storage of a large number of carriers (electrons or holes). In contrast, the resistive switching in a memristor is realized via a limited number of ions migrating through a switching layer or at the electrode/switching layer interface (Box 1), so it is more challenging to quantify and control the ions engaged in the switching process. Meanwhile, the location and morphology of the ion migration region, e.g., conduction filament region, appear to be largely random, governed by a number of currently unknown factors, which in turn exacerbate the parameter variability issue^21^. The situation can be further complicated due to the expansion of active areas by ion migration to new locations during cycling, leading to the irreversible damage to devices^36^. All of the above-mentioned uncertainties and complications can cause the eventual failure of memristive switching. Therefore, from a materials science perspective, understanding ion migration in active materials during resistive switching is crucial for building robust memristors, which is the main focus of this Review (Fig. 1c).

## A synergistic characterization approach

To characterize the localized ion motion in memristors, especially the dynamics during real-time cycling operations, it is essential to combine in situ characterization techniques with device modeling—a synergistic approach—as outlined in Fig. 1c. Using a variety of in situ techniques, the dynamic evolution of electrochemical and structural changes inside the functioning device during resistive switching can be visualized to a certain degree, and some of the dominant dynamic factors can be captured via the direct observations. However, experimental characterization techniques always have certain limitations in observing the switching process due to finite spatiotemporal resolution, which can be complemented and completed by modeling studies. Based on the input of the experimental results, equations describing the dynamic switching process can be developed using appropriate physical modeling, and the driving forces, electron conduction, as well as ion migrations can be simulated at the atomic level. With the combination of in situ techniques and device modeling, a holistic picture of switching mechanism can be eventually established.

**Fig. 2:**
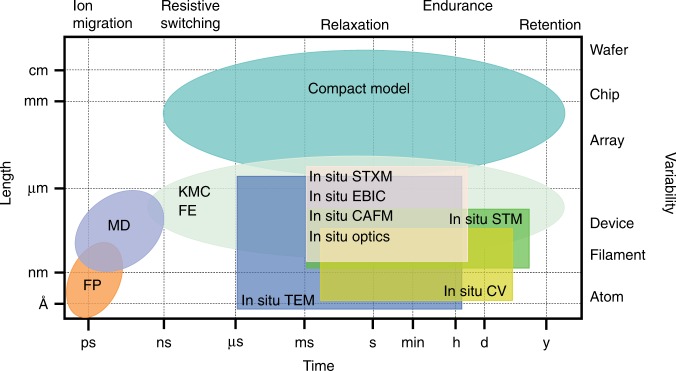
Spatiotemporal resolution of various in situ techniques and device modeling methods. In situ techniques in the rectangular regions: TEM, transmission electron microscopy; CAFM, conductive atomic force microscopy; Optics, optical microscopy; EBIC, electron beam-induced current imaging; STXM, scanning transmission X-ray microscopy; STM, scanning tunneling microscopy; and CV, cyclic voltammetry. Device modeling methods in the elliptical regions: FP, first principles; MD, molecular dynamics; KMC, kinetic Monte Carlo; FE, finite element; and compact modeling. The physical properties and electrical behaviors of memristors are marked according to their corresponding time scale, including ion migration, resistive switching, relaxation, and cycling endurance. The physical presence of memristors on a different level was marked according to the length scale, including filament, device, array, chip, and wafer. The parameter variability of the device performance could be introduced on each level of physical presence. Scale bars provide a visual indication of the spatiotemporal units, not proportional with the actual size

The switching mechanism needs to be studied over a broad spatiotemporal range, which requires a closely collaborative application of in situ techniques and device modeling (Fig. 2 and Table 2). The time scale of ion migration can vary over a range from picoseconds to seconds depending on the condition of the driving force. Due to the fast speed of ion migration under high electric field and/or elevated temperature, resistive switching may occur as rapidly as a sub-nanosecond^37^. Typically, the switching speed is exponentially dependent on the applied voltage^38^. The requirement for data retention in commercialization is normally ten years at ambient temperature. On the other hand, the parameter variability of memristors results from the unstable behavior of the conduction filaments, as well as non-uniformity during fabrications at the device, array, chip, and wafer levels. The diameters of the conduction filaments range from angstroms to nanometers^9,13^. In Fig. 2, the temporal ranges of the physical properties and the electrical behaviors are positioned along the top horizontal axis, and the spatial ranges from atom level to wafer level along the right vertical axis. In principle the combination of in situ techniques and device modeling is capable of covering a broad spatiotemporal range and thus providing a comprehensive understanding of device behavior. The in situ techniques can typically work in a temporal range from microsecond scale to several days and in a spatial range from sub-angstrom level to several micrometers. Device modeling further expand this temporal-spatial region. The first principles (FP), molecular dynamics (MD), and kinetic Monte Carlo (KMC) methods can be used to investigate the resistive switching of memristors down to less than a picosecond, whilst KMC and compact models cover long-time domains up to years.

**Table 2:** Features of various in situ techniques for investigating the switching mechanism

In situ technique	Spatial scale	Temporal scale	Sample type	Information
In situ TEM^100^	Å ~ μm	μs ~ hours	Vertical structure Lateral structure Tip-based structure	Filament morphology Chemical distribution Electronic structure IV measurement
In situ STXM^26,51^	nm ~ μm	ms ~ hours	Vertical structure	Elemental distribution Chemical state IV measurement
In situ EBIC^52^	10 nm ~ 10 μm	ms ~ mins	Vertical structure Lateral structure	Distribution of electric field IV measurement
In situ STM^30^	nm ~ 100 nm	100 ms ~ days	Tip-based structure	Local electron density IV measurement Cycling
In situ CAFM^42^	10 nm ~ 10 μm,	100 ms ~ days	Tip-based structure	Electrochemical reactions IV measurement Cycling
In situ cyclic voltammetry^57,58^	Å ~ 100 nm	10 ms ~ days	Vertical structure	Electrochemical kinetics Diffusion activity
In situ optical microscopy^56^	100 nm ~ 10 μm	s ~ hours	Lateral structure	Filament morphology Electrochemical reactions

The strategy of combining in situ characterization with device modeling has shown a great potential in mechanism understanding and performance optimization of memristors. For instance, Li et al. have used in situ holography to characterize the evolution of oxygen vacancies distribution in a HfO_2_-based memristor under electrical biases^24^. The dynamics of the oxygen vacancies were also modeled by KMC simulation, revealing that the rupture of conduction filaments occurred in the region near the top electrode. Using scalpel scanning probe microscopy (SPM), Celano et al. differentiated the oxygen exchange layer (OEL) from the conduction filament in Hf/HfO_2_ and Ta/Ta_2_O_5_ systems with a nanometer lateral resolution^39^. First principles (FP) calculation indicated that the diffusion of oxygen at the OEL/oxide interface can induce variations in the density of states near the Fermi level in the oxide. The minimum thickness of the OEL/oxide interface has to be ~3 nm to achieve the optimized performance. For a Ag/ZrO_2_ system, Liu et al. discovered that an electric field increased the nucleation of nanocrystals in the ZrO_2_ layer, providing controllable locations and directions for the growth of Ag conduction filaments. The driving force distribution has been simulated by the finite element (FE) methods^40^. These examples showcase that in situ observation techniques provide valuable information on the dominant dynamic factors determining the morphological or compositional changes upon resistive switching, based on which the equations governing the ion migration can be further developed. The developed models can in return guide the understanding of the existing experimental results and the design of new experiments.

## Memristive switching studied by in situ techniques

**Fig. 3:**
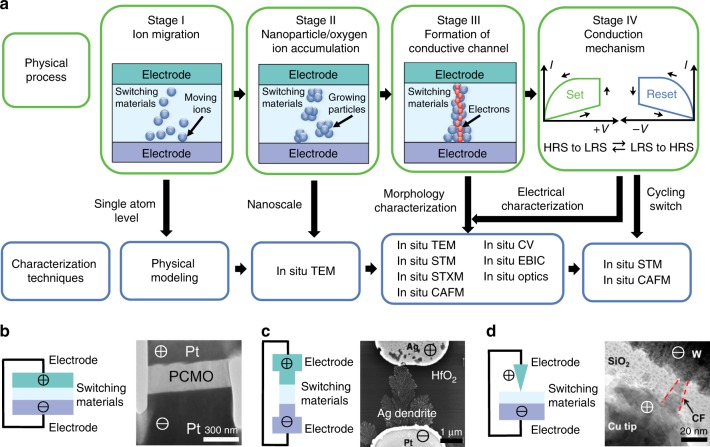
Application of in situ techniques at different switching stages. **a** Technique for characterizing the different physical stages of resistive switching in a memristor. Green boxes are the physical processes for different stages starting from the initial ion migration to the resistive switching between the HRS and LRS. The schematic models for each stage are constructed according to the switching mechanisms of the cation devices, to demonstrate the spatiotemporal scale for each stage. Blue boxes are the corresponding characterization techniques for each physical stage. **b** Schematic and experimental examples of the vertical structure, **c** lateral structure, and **d** tip-based structure. Reproduced from ref. ^43^, Elsevier B.V. (**b**); ref. ^45^, AIP (**c**); and ref. ^46^, American Chemical Society (**d**)

From a microscopic view, the in situ characterization of switching dynamics can be divided into four different physical stages (Fig. 3a). In stage I, the initial step is the generation of mobile ions in the switching layer or at the switching layer/electrode interface, followed by the drift of ions under an electric field. The redox reactions in memristors are essentially electrochemical reactions, which are closely related to the interfaces and moisture concentration in the ambient atmosphere^41^. Although diffusion coefficients have been determined by experimental methods^41^, this dynamic process, from the redox reactions to the nucleation of mobile atoms in the switching layer, has not been directly characterized using existing in situ techniques, partially because of the high temporal resolution required. During stage II, after the aggregation of mobile ions develops into nanoparticles for cation devices or the concentration of oxygen ions reaches a detectable level for anion devices, in situ TEM is the most suitable technique for visualizing the dynamic evolution of morphology at nanoscale and the moving paths of the nanoparticles. Once the formation of conduction region (filamentary or non-filamentary) is completed, the memristor is switched from the high resistance state (HRS) to the low resistance state (LRS) in stage III. The morphological evolution of the ion migration region and the resistive switching of the device can be characterized simultaneously by most of in situ techniques. The correlation between the morphology change and the electric behavior provides clues to parameter variability of memristors. During stage IV, the memristor is switched back and forth between the HRS and LRS by applying switching voltages. To characterize this repetitive cycling, in situ scanning tunneling microscopy (STM) and in situ conductive atomic force microscopy (CAFM) can be employed to monitor local morphological changes in the conduction channel and the resistive switching of the device^30,42^. Therefore, in situ STM and in situ CAFM are key techniques for studying device failure during cycling endurance.

Different device structures have been in situ characterized, including vertical, lateral, or tip-based structures. The vertical structure is a thin slice of the stacked device (Fig. 3b)^43^ or a stack of a three-layer device on a supporting membrane^12^. Such vertical structure more closely represents the actual devices in applications^44^. With the lateral structure (Fig. 3c)^45^, the memristive device is usually fabricated directly on a specialized chip designed for in situ characterization. Since the gap between the two electrodes is wider than the vertical structure, the dynamics of ion migration can be easily observed. The tip-based structure utilizes a metallic tip on the instrument as the opposite electrode to form a memristive device with the oxide layer and metal substrate (Fig. 3d)^46^. In this structure, ion migrations happen around the tip electrode, which is easier for device preparation and provides a better control for the resistive switching during dynamic observations.

**Fig. 4:**
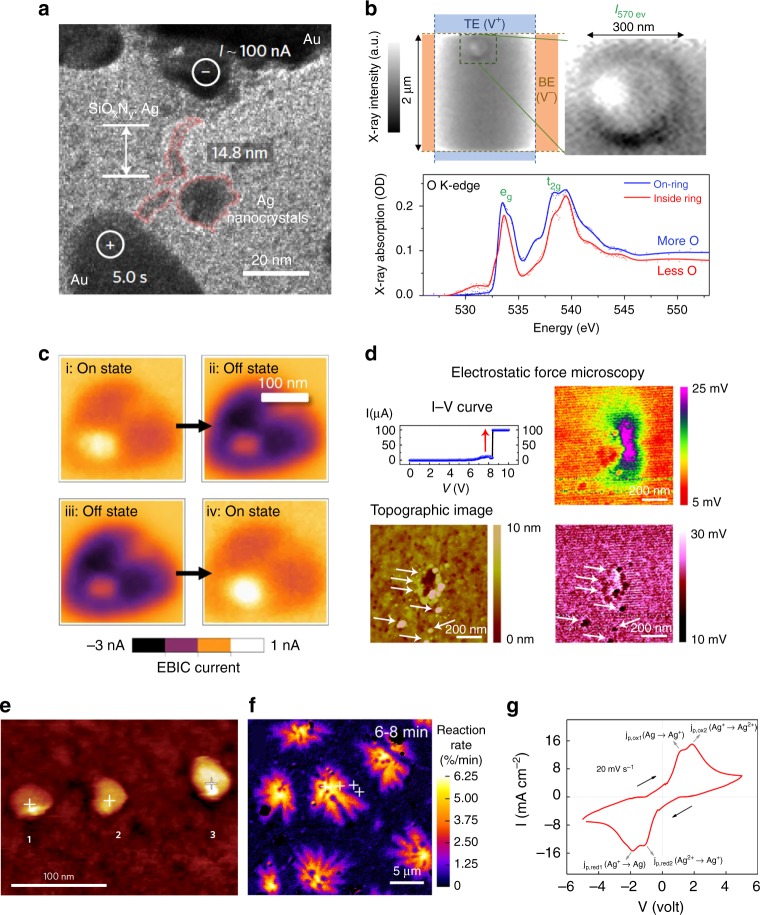
Representative experimental results obtained by in situ techniques. **a** in situ TEM: observation of conduction filaments in diffusive memristors, Au/SiO_x_N_y_:Ag/Au device. **b** in situ STXM: the oxygen concentration difference in a ring-like feature during the resistive switching of HfO_x_ memristors. **c** in situ EBIC: switching micrographs between on and off state in TiO_2_ memristor. **d** in situ CAFM: topography and electrical measurement of the accumulation of oxygen ions in HfO_2_ memristors. **e** in situ STM: highly conductive region in TaO_x_ film. **f** in situ optical microscopy: the redox reaction dynamics in graphene. **g** in situ cyclic voltammetry: redox reaction peak of Ag in Pt/graphene oxide/Ag memristors. Reproduced from ref. ^9^, Macmillan Publishers Ltd (**a**); ref. ^51^, American Chemical Society (**b**); ref. ^52^, Nature Publishing Group (**c**); and ref. ^53^, Nature Publishing Group (**d**); ref. ^30^, Macmillan Publishers Ltd (**e**); ref. ^56^, American Chemical Society (**f**); ref. ^57^, WILEY-VCH Verlag GmbH & Co. KGaA, Weinheim (**g**)

Currently, several in situ techniques have been employed individually to study the switching mechanisms of memristive switching. Table 2 lists the details of their operational features. In situ TEM plays an important role in elucidating the underlying microscopic mechanisms of switching dynamics due to its unprecedented spatial resolution^47,48^. For the cation devices, the formation of a conductive bridge composed of Ag nanoparticles under an applied bias was observed by high-resolution bright-field imaging, under the driving force of electric field and Joule heating (Fig. 4a)^9^. The dynamic relaxation process of the conduction filament was recorded after the removal of biasing. Furthermore, the combined application of in situ TEM and atom probe tomography (APT) has revealed the elemental distribution of conduction filaments in Ag/TiO_2_/Pt devices in three-dimensional space^13^. The results showed that the conduction filament was Ag-doped TiO_2_, which means that the switching dynamic was accompanied by doping/de-doping with mobile ions rather than the connection/disconnection of the entirely metallic conduction filament. For anion devices, the evolution of oxygen ions in a single crystal during resistive switching was identified by the combination of TEM and electron energy loss spectroscopy (EELS)^49^. It was found that the change in the overall oxygen vacancy concentration within the device is associated with the electrocatalytic release and reincorporation of oxygen at the electrode/oxide interface. Although it is challenging to characterize the oxygen ions/vacancies in amorphous materials, the combination of in situ TEM and electro-holography provides a workable solution^24^. The experimental results demonstrated that the conduction filaments constituted by oxygen vacancies were initiated in the bulk of the HfO_2_ layer and ruptured at the interface of the HfO_2_/TiN top electrode^24^.

Besides the significant findings via in situ TEM, switching mechanisms were also partially elucidated via other in situ techniques. In situ scanning transmission X-ray microscopy (STXM) enables nondestructive studies of a vertical specimen to reveal chemical composition distribution^50^. The formation of conduction filaments in Pt/HfO_2_/Hf/Pt devices was captured under biasing, finding a ring-like feature in Fig. 4b^51^. The local distribution of an electronic structure in TiO_2_-based memristors was measured with in situ electron beam-induced current (EBIC), as shown in Fig. 4c, providing a clear image of the underlying switching region, and reveals propagating polarization domains of symmetrical device structures^52^. Electron transport and electrochemical reactions of HfO_2_-based memristors were investigated with in situ CAFM. As shown in Fig. 4d, increasing the applied voltage generated an oxygen-deficient region, and oxygen vacancies accumulated in the location with higher applied bias, leading to the formation of conduction filaments with diameters of ~15 nm^53^. As shown in Fig. 4e, the highly conductive regions in TaO_x_-based memristors were visualized by in situ STM^30^. The measured corresponding *I–V* curve indicated that a purely metallic or mixed metallic-semiconductor structure was formed during resistive switching, suggesting that both cations and anions participate in the switching process in oxide-based memristors^30^. Valov et al. have developed an approach involving the STM technique to visualize the electrochemical formation and dissolution of Ag clusters^54^. Through further material design, a single ion/atom transfer can be manipulated in an atomic layer-by-layer manner, enabling a discrete control of the electrical properties^55^. Combined with in situ TEM, in situ STM-TEM system have been applied successfully to reveal the details of the electrochemical dynamics in cation memristive devices, including the migration and redox reactions of Cu^+^, and the nucleation and growth of Cu protrusion. This finding convincingly demonstrates that the chemical potential difference is a key driving force for the oxidation of Cu, besides the external electric field. The dynamics of the redox reaction for a lateral graphene oxide device was visualized by in situ optical microscopy (Fig. 4f)^56^, presenting effective information of filament growth kinetics. Redox reaction at the interface of the active electrode and the electrolyte was studied with in situ cyclic voltammetry^57,58^. The activation barrier for the reduction (Ag^+^ to Ag, Ag^2+^ to Ag^+^) and oxidation (Ag to Ag^+^, Ag^+^ to Ag^2+^) reactions experienced by Ag atoms/ions at the cathode and anode can be measured (Fig. 4g)^57^, and a second-order oxidation of Ag at the interface was observed.

In summary, most in situ characterization results have been focusing on the formation of conduction filaments (stage III) and the corresponding resistive switching, while the initial ion migration (stage I), nanoparticle movement (stage II), and cycling between LRS and HRS (stage IV) have been less often revealed using in situ techniques. However, it has become more and more clear that the parameter variability of memristive devices is closely related to the non-uniformity of the conduction path during set process and the variation of the gap length after the rupture of conduction path during reset process. Unfortunately, the corresponding details remain poorly understood to date, while the factors determining cycling endurance haven’t yet been well investigated either. Therefore, an immediate attention to the switching mechanism in stages I, II, and IV via in situ studies is urgently demanded.

## Device modeling based on in situ characterizations

Device modeling and in situ techniques complement each other to improve our understanding of resistive switching. According to different application requirements, device modeling can be classified into physical modeling and compact modeling (Box 2). One target of physical modeling is to reconstruct the ion migration during the resistive switching process. For example, while in situ techniques are capable of characterizing the morphology of conduction filaments as shown in Fig. 1c, physical modeling can be further used to shed light on the intermediate filament growing/rupturing processes at high spatial and temporal resolutions beyond those of experiments^59–61^. Modeling results can also clarify the driving force by simulating the distribution of electric field, temperature, and chemical potential in the active layers^61–63^.

**Fig. 5:**
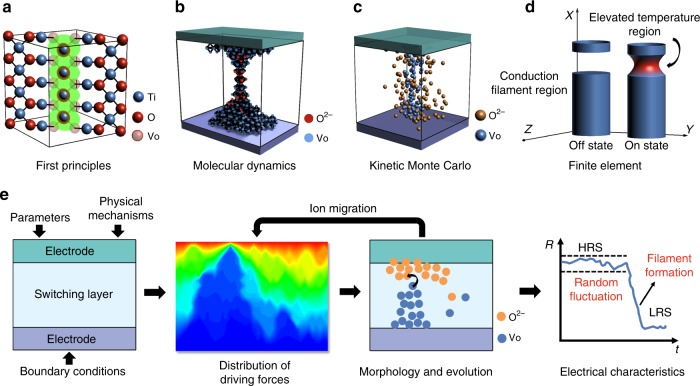
Strategy for physical modeling of a memristor. **a** Schematic of the FP method to calculate the property of oxygen vacancies. **b** Schematic of the MD method to simulate the ionic migration dynamics. **c** Schematic of the KMC method to simulate the distribution of oxygen vacancies and interstitial ions. **d** Schematic of the FE method to calculate the morphology of conduction filaments. **e** Flow chart of the suggested complete flow of physical modeling and numerical simulation on memristive devices. First the related physical equations and parameters are considered in the MIM structure. Then and distribution of driving force and ion motion in the switching layer are calculated. These calculations are repeated for many times as the whole switching process evolves multiple ion motion events. Finally, the electrical behaviors (e.g. variability and switching) of the memristor is output based on the microscopic simulation and electron transport modeling

Several physical modeling techniques have been explored for this purpose. First Principles (FP) (also called ab initio) calculations are widely used to obtain the conduction property of a stable state and the transition energy between different stable states^63^. As illustrated in Fig. 5a, FP calculations were used to construct a supercell with tens to hundreds of atoms. With this technique, there is no need to introduce any model parameters. A typical task with the FP approach is to calculate the activation barrier of ion migration. This calculation is very useful for evaluating whether a specific doping configuration can help improve the uniformity or reliability of a memristor^64^. FP calculations have also been used to investigate some simple dynamics, such as single oxygen migration events, charging/decharging events^63,65^, or resistance volatility^66^. However, existing FP approaches still face the challenge of how to accurately model the polycrystalline, amorphous, or complex stack structures in real memristive devices. In addition, it is also challenging to simulate long-term and multi-event dynamic processes owing to its complex computation procedure.

Dynamic simulation techniques, such as MD^67^ (Fig. 5b) and KMC^59^ (Fig. 5c), are powerful tools for studying the ion migration process in memristor and related variabilities. A reactive MD with the charge equilibration method was proposed to investigate the formation of metastable atom-chain and stable filaments in the cation memristor^67^. KMC is essentially a stochastic and event-driven method. It does not need to perform calculations for every time period; instead, it counts only the duration between two events. Thus, it is easy to cover a broad time scale ranging from resistive switching over nanoseconds to long-term retention over years^59,68^. To model the ion distribution accurately, the KMC method is applied in combination with the finite element (FE) method (Fig. 5d). Whilst, the FE method is sometimes used separately to simulate the resistive switching without considering the parameter variability^69^. In this case, only the concentration of oxygen vacancies is calculated, as illustrated in Fig. 5d, thus simplifying the simulation. The disadvantage of the KMC and FE methods is that they require many model parameters and assumptions.

Another goal of physical modeling is to connect the microscopic ion distribution with the macroscopic electrical performance. In some cases, the electron transport mechanisms of memristors can directly fit in the macroscopic model (Table 1). However, with complex structural and material components, memristors may exhibit unique *I*–*V* curves that cannot be fit well with existing models. In this case, the modeling of *I*–*V* characteristics should start from the microscopic mechanism correlated with a specific distribution of ions. A simplified way to model *I*–*V* characteristics is to use a resistor network. The resistors can vary according to the corresponding oxygen vacancy distribution^62^. A more general way is to solve the Poisson equation and current continuity equation.

Figure 5e summarizes a more complete flow to model and simulate resistive switching processes based on the approaches reported previously^59–63^. First, decisions should be made on which physical mechanisms are taken into account and how to quantify these physics processes with appropriate equations. Typically, most models start from ion migration or oxygen vacancy generation based on the defect theory. The parameters should be input based on the experimental extraction or FP calculations, and the boundary conditions should be fixed. Subsequently, the distributions of various driving forces are calculated, and a dynamic method is used to describe the ion migration. With an updated ion distribution, the dynamic switching processes including microscopic morphology evolutions and macroscopic electrical characteristics are finally simulated.

Compact modeling also plays a crucial role for memristor research, especially in circuit analysis. Unlike CMOS transistors, the compact model of memristors should not only focus on the basic electrical characteristics but also capture the parameter variability and cycling endurance degradation characteristics. Typically, to construct a compact model of memristor, the basic model framework is firstly developed using a simplified physical image and analytical equations. Then, the degradation parameters and randomness parameters are introduced in the framework to describe the cycling endurance and parameter variability characteristics^70–72^. After that, the model parameters are extracted based on the statistical measurements from amount of memristive devices and repetitive cycling tests. Finally, a SPICE model (simulation program with integrated circuit emphasis model) can be constructed by further extracted the parameters of parasitic circuit elements. To ensure the accuracy of circuit simulation, the compact model of memristors also calls for a complete picture of switching mechanism, since most of the compact models are physics-based.

Box 2 Classification of memristive device models Based on different application requirements, device modeling of memristor can be classified into different levels. Physical modeling starts from microscopic physical processes of resistive switching, using numerical simulation approach to accurately reproduce to device behaviors^59,60^. Physical modeling aims to clarify switching mechanisms in complementary with experimental characterizations and provide guidelines for device performance optimization^61^. Compact modeling introduces some empirical assumption without concerning the underlying physics. Compared to physical modeling, compact modeling provides the possibility of rapidly reproducing the phenomenological electrical behavior of memristors with a low computation cost. Some compact models are established by mathematically fitting the measured electrical behaviors of memristors^85,86^, which are also called behavioral models. The behavioral models are highly simplified and easy to extend to different types of memristors; however, they cannot capture the variabilities in a real circuit. For example, in a crossbar array, the voltage drop on each memristor cell is different because of the interconnected resistance^70,87^, therefore, the resistive switching behaviors are different for each cell, which is almost impossible to be captured by the behavioral model. Therefore, the behavioral models are mainly suitable for system-level simulations, such as the benchmarking of a memristor-based neuromorphic network^71^. On the other hand, a physics-based compact model is more useful for circuit-level simulation. Most physics-based compact models of memristors are derived using the FE method by quantifying the morphological or compositional changes with simplified physical equations^88,89^. The simulation program with integrated circuit emphasis (SPICE) model is a special type of compact model. It should be analytical so that it can be easily embedded into the commercialized technology computer-aided design tools. A good SPICE model for a real memristive device should include the parasitic circuit elements, so that it can be accurate for the simulation of a complex mixed memristor/CMOS circuit^87,90^. A physics-based model can help to understand the device mechanism while a purely behavior model does not.

Schematic for the device modeling of memristors: **a** Physical modeling. **b** Memristive device design with a physical model. **c** Compact modeling. **d** Crossbar array simulation using a compact model. **e** SPICE modeling. f Memristor/CMOS mixed circuit simulation using a SPICE model. Reproduced from ref. ^91^, Nature Publishing Group (**b**); ref. ^72^, IEEE (**c**); ref. ^87^, IEEE (**e**).

## Outlook and perspectives

Despite recent progress in the in situ characterization and device modeling of resistive switching, there are still many aspects not fully understood yet. The currently available data from in situ measurements has only uncovered a small portion of the entire picture of memristive switching, which in turn limits the application of device models to further understand it. This situation thus calls for in situ characterizations of memristors at high resolution in three dimensions (3D) and physical modeling with high degree of accuracy capable of providing practical guidelines for device optimization in the future. To this end, here we propose several research directions that are crucial to tackle the challenges.

First, we need to continuously improve the observation resolution in order to monitor the dynamics of ion migration in a region of atomic distances and sub-ns intervals. Currently the biggest obstacle to increase spatial resolution is the mobility of anions migrating through amorphous oxide layers, making it extremely challenging to identify their positions. One possible solution is the use of isotope elements to trace the movement of ions^73^. The switching dynamics occurring in a short time range of nanoseconds to milliseconds can be investigated via ultrafast transmission electron microscopy (UTEM)^74^. Another plausible approach is to reduce the speed of resistive switching using low temperatures and to characterize the dynamic evolution using cryo-TEM techniques^53^.

Secondly, the characterization of switching dynamics needs to be conducted at higher dimensions. The 3D characterization of conduction filaments is expected to provide a complete representation of their morphology^13,42^. A 4D evolutionary representation that demonstrates the 3D switching dynamics over time can provide more direct information for understanding the switching mechanisms. In situ techniques that can be applied in this aspect include electron tomography^75^, 3D STEM tomography^76^, and 4D UEM^74^. Studying the influence of environmental conditions, such as different ambient atmosphere and thermal field, can not only provide additional evidence of switching mechanism, but also help to evaluate the potential of memristors for some special applications, like automotive electronics. Environmental TEM^77^ is capable of providing a simulated environment with a controlled humidity, gas composition, liquid mixture, and temperature.

Finally, we should continuously use device modeling to further understand the switching mechanism, especially beyond the resolutions of the characterization techniques, and thus possibly use the insights obtained from modeling to guide device optimization and design. With the help of predictive models, we could be able to understand how to best combine materials or design device architecture to improve device uniformity and reliability. We can also use the modeling to investigate the theoretical limit of the minimal cycle-to-cycle and device-to-device variation in addition to the maximum cycling endurance, based on which we decide on how to cope with this non-ideal performance at a circuit level. The accuracy of memristor models needs to be continuously improved. It is essential to develop realistic physical models for the amorphous and polycrystalline states of active materials to accurately reproduce that in testing devices. The combination of percolation theory and Monte Carlo simulation provides a possible solution to model the ion migration and electron transport in an amorphous system. To capture the variability and endurance degradation over trillions of switching cycles, it is recommended to utilize machine learning in the simulation of memristors^78,79^. After training a neural network with the pre-calculated electrical data in a few switching cycles via device modeling, a neural network can fast predict the data during the following cycles, no need to repeat the time-cost atomic-scale simulation.

In view of all of above-mentioned opportunities, it is our hope that large parameter variability and poor cycling endurance issues that we are currently encountering in memristor research can be eventually solved based on a comprehensive understanding of the resistive switching mechanism. To this end, one possible approach is to design material stacks with predictable ion migrating paths by confining the formation/rupture of conduction filaments to local regions using nano-fabrication technologies, such as deliberated doping, multilayer and side-wall protected structure, capping layer modulation, etc.

In conclusion, we have reviewed the current research status of the switching mechanism of memristors, outlined a synergistic strategy of combining in situ techniques and device modeling to uncover the detailed switching mechanism and examined its potential. We advocate immediate action to further investigate memristive switching dynamics at higher resolutions and higher dimensions in experiments, and a larger degree of accuracy in modeling. Continued research efforts are required to solve the practical issues associated with parameter variability and cycling endurance of memristive devices before they could be possibly transferred to commercialization for future memory and computing technologies.
